# High-fat, high-sugar diet induces splenomegaly that is ameliorated with exercise and genistein treatment

**DOI:** 10.1186/s13104-018-3862-z

**Published:** 2018-10-22

**Authors:** Levi Buchan, Chaheyla R. St. Aubin, Amy L. Fisher, Austin Hellings, Monica Castro, Layla Al-Nakkash, Tom L. Broderick, Jeffrey H. Plochocki

**Affiliations:** 1 Arizona College of Osteopathic Medicine, Midwestern University, Glendale, AZ USA; 2 College of Graduate Studies, Midwestern University, Glendale, AZ USA; 3 Department of Anatomy, College of Graduate Studies and Arizona College of Osteopathic Medicine, Midwestern University, Glendale, AZ USA; 4 Department of Physiology, College of Graduate Studies and Arizona College of Osteopathic Medicine, Midwestern University, Glendale, AZ USA; 5 Department of Physiology, Laboratory of Diabetes and Exercise Metabolism, College of Graduate Studies and Arizona College of Osteopathic Medicine, Midwestern University, Glendale, AZ USA; 6 Department of Medical Education, College of Medicine, University of Central Florida, 6850 Lake Nona Blvd, Orlando, FL 85308 USA

**Keywords:** High-fat diet, High-sugar diet, Spleen, Exercise, Genistein

## Abstract

**Objective:**

We tested the effect of exercise training and genistein treatment on splenomegaly in mice fed a high-fat, high-sugar diet (HFSD).

**Results:**

Male and female C57BL6 mice fed HFSD containing 60% fat along with drinking water containing 42 g/L sugar (55% sucrose/45% fructose) for 12 weeks exhibited significant obesity, hyperglycemia, and elevated plasma IL-6 levels. This was accompanied by splenomegaly characterized by spleen weights 50% larger than mice fed standard chow (*P* < 0.05) with enlarged rad and white pulps. Mice fed HFSD and treated with a combination of exercise (30 min/day, 5 days/week) and genistein (600 mg genistein/kg diet) had reduced spleen weight (*P *< 0.05). The decrease in spleen weight was associated with a significant improvement in red-to-white pulp area ratio and plasma glucose and IL-6 (*P* < 0.05). Our findings indicate that reversal of splenomegaly by regular exercise and genistein treatment may be important in the clinical management of HFSD-induced obesity.

## Introduction

Obesity, type 2 diabetes mellitus, and other metabolic disorders are being reconceptualized as inflammatory conditions [1, 2]. For example, obesity induced by high-fat, high-sugar diet (HFSD) is associated with prolonged elevation of proinflammatory serum markers such as IL-6 and inflammation in peripheral tissues, as well as metabolic dysregulation, including insulin and leptin resistance [3, 4]. Although the effects of diet-induced metabolic dysregulation and inflammation are widely documented in many organs, its effects on spleen morphology have yet to be thoroughly characterized.

The spleen is the largest secondary lymphoid organ in the body and is composed of two functional regions, white and red pulp. White pulp is lymphoid tissue containing immune cells that target blood-borne pathogens, whereas red pulp is a site of erythrophagocytosis [5, 6]. Inflammation induced by HFSD has been shown to modulate splenic function by causing increased phosphatidylserine externalization of red blood cells and thus promoting the interaction with erythrophagocytosis macrophages [6], and by inducing extramedullary hematopoiesis of monocyte-like cells secondary to inflammation [7]. These changes in splenic function and morphology have been implicated in the pathogenesis of diabetes and obesity-related cardiovascular disease and kidney disease [6, 8]. Therefore, therapeutic modalities that maintain normal splenic morphology in the obese condition may prove beneficial to long-term health.

In this study, we examine metabolic and proinflammatory markers, spleen weight, and spleen histomorphometry in mice fed a HFSD and treated with either exercise or the isoflavone genistein, or both. Treatment with exercise and isoflavones have been shown to ameliorate peripheral inflammation through antioxidative actions and by reducing levels of proinflammatory cytokines [3, 9, 10]. In this study, we hypothesize that exercise and genistein treatment in mice fed a HFSD mitigates diet-induced changes in spleen weight and morphology.

## Main text

### Methods

Fifty female and 50 male mice of the strain C57BL6 (Jax Labs, ME, USA) were used in the study. At the age of 6 weeks, the mice were randomly divided into 5 treatment groups of 10 mice per sex. Treatment groups were assigned as follows: (1) untreated control mice, (2) mice fed a HFSD, (3) mice fed a HFSD and treated with exercise, (4) mice fed a HFSD and treated with genistein, (5) and mice fed a HFSD and treated with exercise and genistein. Treatment was administered for 12 weeks. Mice in the HFSD groups were fed pellets with 60% fat, 20% protein and 20% carbohydrate (Dyets Inc. Bethlehem, PA, USA) and given 42 g/L sugar dissolved in drinking water (55% fructose/45% sucrose). This diet induces significant visceral obesity and insulin resistance in the C57BL/6 mouse [11]. Control mice were given standard drinking water and rodent chow that contained 20.3 g protein, 66 g carbohydrate, and 5 g fat. All foods and liquids were administered ad libitum. Exercise treatment consisted of low-intensity treadmill running for 30 min/day, 5 days/week. Exercise of this duration and intensity was chosen because it has been shown to reduce insulin resistance in C57BL/6 mice with diet-induced obesity [12]. Genistein treatment was administered at 600 mg genistein/kg HFSD diet (Dyets Inc., PA, USA). We have previously found this genistein dose incorporated into diet is sufficient to produce significant increases in free genistein in plasma and to benefit bone and gut health [13, 14]. During the study, mice were housed at a temperature of 22 °C with a light/dark period of 12-h. Use of the animals was approved by the Institutional Animal Care and Use Committee at Midwestern University. The protocol of the experiment complied with the National Institutes of Health’s Guide for the Care and Use of Laboratory Animals.

Following sacrifice at an age of 4 months, spleens were harvested, weighed, and embedded in paraffin blocks. Spleens were then sectioned longitudinally in the midline at 5 μm thickness and stained with hematoxylin and eosin (H&E) for histological evaluation under light microscopy. ImageJ (v1.6, NIH) was used to measure the area of the spleen and the ratio of white to red pulp, calculated as [(red pulp area − white pulp area)/white pulp area]. Enlargement of one or both of these splenic regions may indicate dysfunction. Plasma was collected for measurement of glucose (Autokit, Wako Diagnostics, Richmond, VA, USA), insulin, and IL-6 (Milliplex Assay, Millipore, Billerica, MA, USA) following the manufacturers’ instructions. Two-way ANOVA was used to test for differences among the treatment groups and between females and males. Significance was set at *P* < 0.05. Statistical analyses were conducted using SPSS Statistics 25 software (IBM, USA).

### Results

**Fig. 1:**
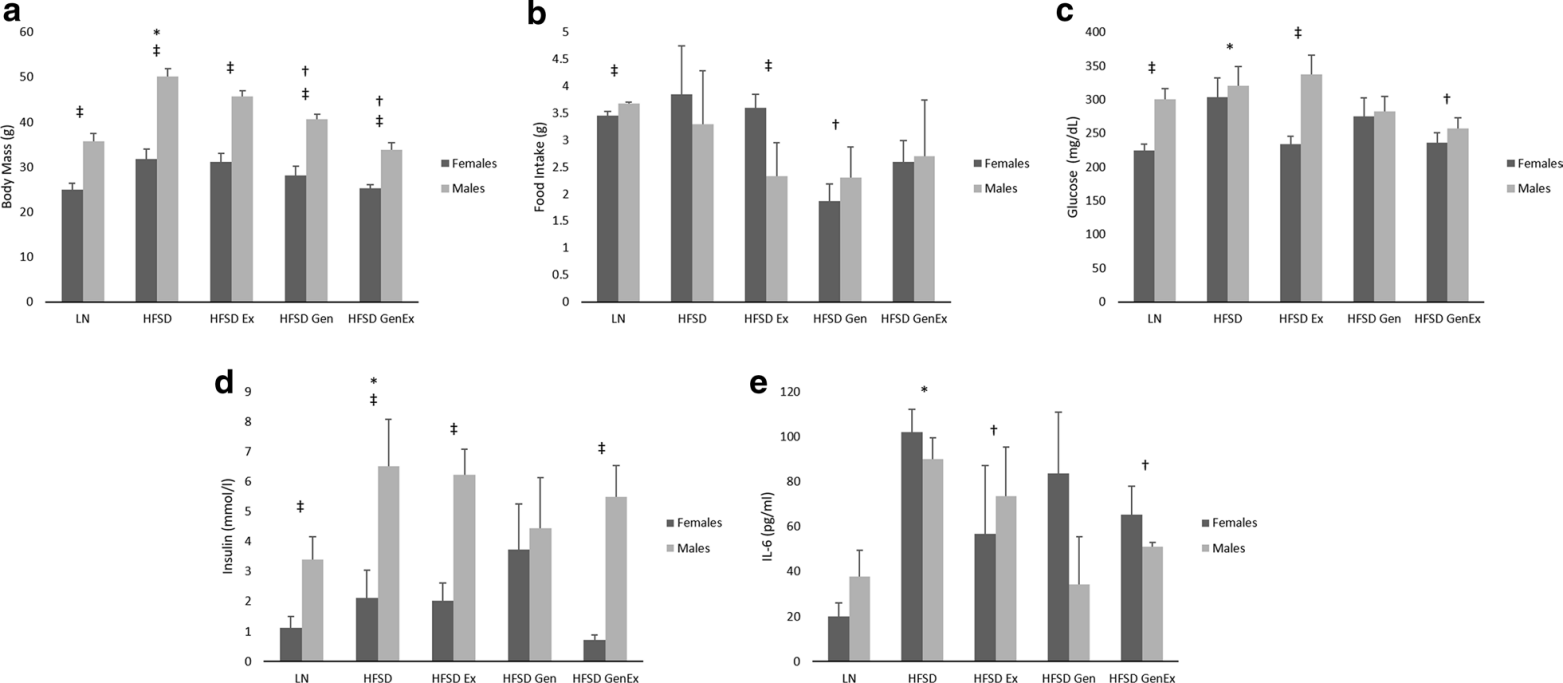
Body mass (**a**), food intake (**b**), and plasma levels of glucose (**c**), insulin (**d**), and IL-6 (**e**) by treatment group. *Significant difference between lean mice fed standard diet and mice fed HFSD (P < 0.05); ^†^significant difference with mice fed HFSD (P < 0.05); ^‡^significant difference between males and females given the same treatment (P < 0.05). LN, lean mice fed standard diet (n = 10 females, 8 males); HFSD, high-fat, high-sugar diet (n = 9 females, 9 males); Ex, exercise (n = 9 females, 10 males); Gen, genistein (n = 8 females, 8 males); GenEx, genistein and exercise (n = 10 females, 8 males). Data are expressed as mean ± 2 SE

Food intake measured by weight was similar in mice fed HFSD and lean mice fed a standard diet (*P* > 0.05), yet mice fed HFSD had significantly greater body mass than lean mice (*P* < 0.05, Fig. 1). Mice fed HFSD and treated with genistein had reduced food intake and body mass in comparison to mice fed HFSD alone (*P* < 0.05). Analysis of plasma markers showed mice treated with HFSD had elevated glucose, insulin, and IL-6 in comparison to lean mice fed standard diet (*P* < 0.05, Fig. 1). Treatment with exercise and genistein in combination reduced plasma glucose and IL-6 in mice fed HFSD (*P* < 0.06). When comparing males and females within each treatment group, we found males fed a standard diet had greater body masses, food intake, and plasma glucose and insulin levels than females given the same diet (*P* < 0.05). Male mice also had higher body mass and insulin levels than females in every treatment group except HFSD + genistein (*P* < 0.05).

**Fig. 2:**
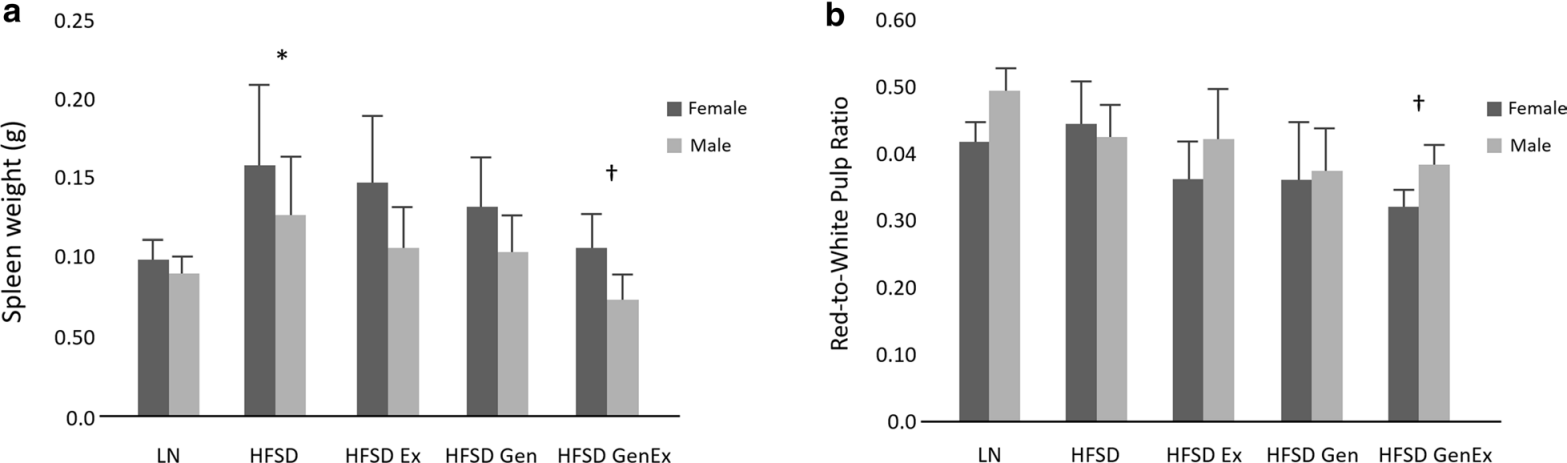
Analysis of spleen weight (**a**) and ratio of red pulp area to white pulp area of the spleen (**b**) by sex and treatment group. As the ratio approaches 0.0, there is greater white pulp area relative to red pulp area. *Significant difference between lean mice fed standard diet and mice fed HFSD (P < 0.05); ^†^significant difference with mice fed HFSD (P < 0.05). LN, lean mice fed standard diet (n = 10 females, 8 males); HFSD, high-fat, high-sugar diet (n = 9 females, 9 males); Ex, exercise (n = 9 females, 10 males); Gen, genistein (n = 8 females, 8 males); GenEx, genistein and exercise (n = 10 females, 8 males). Data are expressed as mean ± 2 SE

**Fig. 3:**
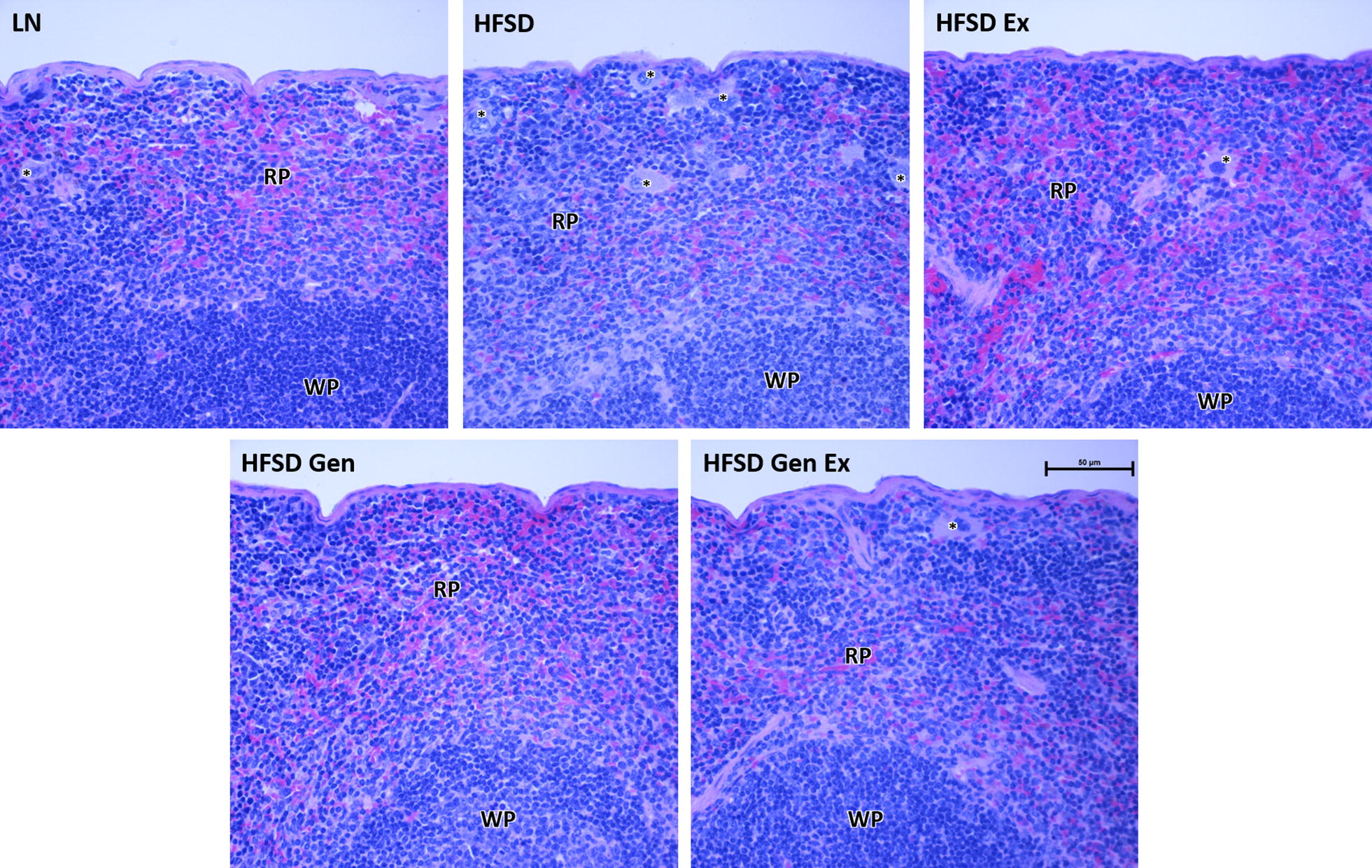
Representative histological sections of the spleen for each treatment group. Note the high cellularity of the control HFSD-fed mice, which is not present to the same extent in the HFSD treated with exercise and/or genistein. Mice fed HFSD also have numerous macrophages in the red pulp (asterisks) in comparison to the other treatment groups. Splenic morphological appearance did not differ by sex. LN, lean mice fed standard diet (n = 10 females, 8 males); HFSD, high-fat, high-sugar diet (n = 9 females, 9 males); Ex, exercise (n = 9 females, 10 males); Gen, genistein (n = 8 females, 8 males); GenEx, genistein and exercise (n = 10 females, 8 males). RP, red pulp; WP, white pulp. Histological analysis was conducted on all 100 mice. H&E stain. Scale bar 50 μm

Comparisons of spleen weights showed mice fed HFSD had significantly enlarged spleens relative to lean mice (*P* < 0.05, Fig. 2). However, there was no difference in red-to-white pulp ratio between these treatment groups, indicating the increase in splenic weight in HFSD mice is due to expansion of both the red and white pulps (*P *> 0.05, Fig. 2). Mice fed HFSD and treated with both genistein and exercise had reduced spleen weight and red-to-white pulp ratios in comparison to mice fed HFSD alone (*P* < 0.05). Male and female mice fed HFSD responded similarly to treatment with exercise and genistein alone and in combination (Fig. 2). Microscopic examination of the spleens found increased cellularity in the red pulps of mice fed a HFSD in comparison to mice fed a standard diet and HFSD mice treated with exercise and/or genistein (Fig. 3). The red pulps of mice fed HFSD contained numerous macrophages, which were not present to the same extent in mice of the other treatment groups (Fig. 3).

### Discussion

Our results show that HFSD significantly alters splenic morphology. Mice fed a HFSD exhibited significant splenic enlargement in comparison to control mice after 12 weeks of treatment. Given that 6 weeks of high-fat diet administration in rats does not significantly increase spleen weight [15], our findings suggest doubling the treatment period or the addition of sugar to the diet may be needed to induce splenomegaly. We found no significant difference in the ratio of red-to-white pulp area in mice fed a HFSD. This suggests splenomegaly is likely attributed to concomitant morphological changes in both the red and white pulp. Altukkaynak et al. [16] found treatment with high fat diet causes sinusoids and surrounding tissue to expand in both the red and white pulps, rather than finding histological changes specific to one pulp. This is consistent with our microscopic observations of the spleen. We further observed numerous macrophages adjacent to the sinusoids of mice fed a HFSD. While HFSD has yet to be studied in the spleen, administration of a high fat diet to mice has been shown to enhance erythrophagocytosis by macrophages [6]. Our observation of increased macrophage presence in the spleen indicates this also occurs with HFSD.

A central goal of our study was to determine the therapeutic value of exercise and the isoflavone genistein on reducing splenomegaly. We show treatment with exercise and genistein in combination inhibits the formation of splenomegaly in mice fed a HFSD. Genistein inhibits angiogenesis by inhibiting proliferation of endothelial cells [17]. It is possible that the anti-angiogenic effects of genistein prevent sinusoidal dilation in the spleen, thereby ameliorating splenomegaly associated with HFSD. This has been hypothesized as an explanation for how genistein treatment reduces splenomegaly in mice with malaria-infected red blood cells, and may be acting in a similar manner here [18]. Genistein also blocks the ingestion of RBCs by macrophages through its actions as a tyrosine kinase inhibitor [19]. Erythrophagocytosis is a major cause of splenomegaly and interruption of this process in the red pulp by genistein may help explain its influence on spleen weight in our study. It may also help explain why the red-to-white pulp ratio is decreased in mice fed HFSD and treated with genistein and exercise. However, additional research is required to explicate the precise mechanism of the contribution of genistein to splenomegaly prevention.

Splenic volume has been reported to decrease in volume during exercise, likely from contractile reticular cells within the splenic stroma [20, 21]. However, this change appears to be transient, although long-term data are lacking [22]. It is perhaps more likely that the contribution of exercise to the observed decrease in spleen weight is associated with reductions in obesity-related inflammation. Exercise is protective against central obesity and insulin resistance, and is associated with a reduction in proinflammatory serum markers [23]. Exercise modulates the function of immune cells that are abundant in the spleen, namely lymphocytes and macrophages [24, 25]. Even light exercise is sufficient to reduce circulating proinflammatory cytokines like TNF secreted by lymphocytes and macrophages [26, 27]. Alterations in immune cell function, particularly in the obese condition [28], may contribute to reductions in spleen volume.

The efficacy of treatment with a combination of genistein and exercise in the HFSD-fed mice on splenomegaly is further corroborated by reductions in body mass and plasma glucose and IL-6 levels also identified in this treatment group. These results suggest genistein and exercise in combination improve metabolic function and inhibit inflammation systemically. Treatment with genistein and exercise in combination may mitigate splenomegaly by beneficially affecting glucose and IL-6 pathways. Glucose intake induces oxidative stress at the cellular and molecular levels that causes inflammation through secretion of IL-6 [29], a proinflammatory marker associated with the obese condition and complications such as cardiovascular disease [30, 31]. Cells incubated with genistein exhibit decreased IL-6 production [32, 33]. IL-6 production is also modulated by glucose cellular uptake during exercise [34, 35]. The combined effects of genistein and exercise on glucose uptake and IL-6 expression may be responsible for the reduction in spleen weight noted in the current study, and may likely have benefits beyond the spleen. However, additional data are needed to elucidate the precise effects of genistein and exercise treatment in the HFSD-induced obese condition.

In summary, this study presents novel findings that augment the current understanding of how splenic morphology is influenced by diet. We show that, (1) HFSD administered over a 12-week period is sufficient to cause splenomegaly in mice, (2) combined exercise and genistein treatment may reverse splenic enlargement associated with HFSD, and (3) the reduction in splenomegaly with combined exercise and genistein treatment directly correlates with reductions in plasma glucose and IL-6 levels. These findings may have implications for the treatment of inflammation and splenomegaly associated with HFSD diet.

## Limitations

Our study is limited in several ways. To better define changes to splenic pulp cellular composition, immunohistochemistry should be conducted to identify proliferation in immune cell populations. Red blood cell labeling in conjunction with a splenic phagocytosis assay would also better inform our understanding of alterations in erythrophagocytosis for each treatment. Interpretations of our findings should be qualified by these limitations.

## Abbreviations

HFSD: high-fat, high sugar diet
Ex: exercise
Gn: genistein
RP: red pulp
WP: white pulp
